# Sedative drugs modulate the neuronal activity in the subthalamic nucleus of parkinsonian patients

**DOI:** 10.1038/s41598-020-71358-3

**Published:** 2020-09-03

**Authors:** Amit Benady, Sean Zadik, Dan Eimerl, Sami Heymann, Hagai Bergman, Zvi Israel, Aeyal Raz

**Affiliations:** 1 St George’s University of London Medical School, Sheba Medical Center, Ramat Gan, Israel; 2 Center of Advanced Technologies in Rehabilitation, Sheba Medical Center, Ramat Gan, Israel; 3 Department of Anesthesia, Hadassah Hebrew University Medical Center, Jerusalem, Israel; 4 Department of Medical Neurobiology, Hebrew University – Hadassah Medical Scholl, Jerusalem, Israel; 5 Department of Neurosurgery, Hadassah Hebrew University Medical Center, Jerusalem, Israel; 6 Department of Anesthesiology, Rambam Health Care Center affiliated with the Ruth and Bruce Rappaport Faculty of Medicine, Rambam Health Care Campus, Technion – Israel Institute of Technology, 8 HaAliya HaShniya St., 3109601 Haifa, Israel

**Keywords:** Parkinson's disease, Neurophysiology

## Abstract

Microelectrode recording (MER) is often used to identify electrode location which is critical for the success of deep brain stimulation (DBS) treatment of Parkinson’s disease. The usage of anesthesia and its’ impact on MER quality and electrode placement is controversial. We recorded neuronal activity at a single depth inside the Subthalamic Nucleus (STN) before, during, and after remifentanil infusion. The root mean square (RMS) of the 250–6000 Hz band-passed signal was used to evaluate the regional spiking activity, the power spectrum to evaluate the oscillatory activity and the coherence to evaluate synchrony between two microelectrodes. We compare those to new frequency domain (spectral) analysis of previously obtained data during propofol sedation. Results showed Remifentanil decreased the normalized RMS by 9% (*P* < 0.001), a smaller decrease compared to propofol. Regarding the beta range oscillatory activity, remifentanil depressed oscillations (drop from 25 to 5% of oscillatory electrodes), while propofol did not (increase from 33.3 to 41.7% of oscillatory electrodes). In the cases of simultaneously recorded oscillatory electrodes, propofol did not change the synchronization while remifentanil depressed it. In conclusion, remifentanil interferes with the identification of the dorsolateral oscillatory region, whereas propofol interferes with RMS identification of the STN borders. Thus, both have undesired effect during the MER procedure.

**Trial registration**: NCT00355927 and NCT00588926.

## Introduction

Deep brain stimulation (DBS) is the treatment of choice for many patients with advanced Parkinson’s disease (PD)^1,2^. The efficacy of this treatment depends on optimization of the locations of the stimulation electrodes in the target nucleus—the Subthalamic Nucleus (STN). For accurate implantation of the DBS electrode into the posterior-dorso-lateral (motor domain) STN, electrophysiological mapping of the nucleus using microelectrode recording (MER) is often employed^3^. The borders of the STN can be identified by recording the typical neural activity of the STN and its neighboring structures^4,5^. In medical centers employing MER, the patient is often awake, off anti-PD medication and with his/her head immobilized and fixated in a stereotaxic frame. Even if a frameless technique is used, the patient’s motion throughout the procedure is limited. Thus, patients may experience discomfort and anxiety during the operation^6^. Despite this, many centers choose not to use sedation during the procedure, out of concern that it may limit the quality of the MER data^7^. A recent review on the subject stated that the consensus remains to minimize anesthetics usage, though the literature is sparse^8^.

Synchronized beta band (13–30 Hz) oscillations are often observed in the dorso-lateral region of the STN of PD patients and are thought to play a role in the disease pathophysiology^4,9^. Beta oscillations play a role in normal motor activity^10,11^. However, synchronized beta oscillations in the STN correlate with worsening of PD motor symptoms^12–14^ and beta power is suppressed by dopaminergic medications and by DBS in proportion to clinical improvement^15,16^. The existence of oscillatory activity at the electrode site can predict the effectiveness of DBS treatment^1^. One hypothesis regarding the mechanism of action of DBS, is that it de-synchronizes abnormal basal ganglia beta oscillations^2,13,15^.

In a previous study, we found that propofol depresses the neuronal activity in the STN and the signal to noise ratio utilized to identify the nucleus (i.e. the ratio of the RMS in the STN to the internal capsule (white matter) RMS). These features return to baseline shortly after the administration of propofol is stopped^17^. Others have reported using remifentanil successfully during MER guided DBS procedure, alone or in combination with other drugs^6,18–20^. However, the effects of remifentanil on the activity of STN neurons and the quality of MER based demarcation of STN borders and domains is not clear yet. We therefore studied the effects of remifentanil on STN neuronal activity during DBS procedure.

## Materials and methods

All study procedures were carried out in accordance with the relevant guidelines and regulations including the Israeli law and the declaration of Helsinki. The study was approved by the Hadassah Medical Organization Institutional Review Board (Ref. code 39–4.8.06 and 22–02.11.07 for the propofol and remifentanil, respectively) and the Israeli ministry of health (Ref. code: 3,769). A written informed consent was obtained from all patients prior to enrolling in the study. The trial was registered prior to patient enrollment at clinicaltrials.gov (NCT00355927, principal investigator: Eimerl D., date of registration: July 25, 2006 for the propofol, and NCT00588926, principal investigator: Eimerl D., date of registration: January 9, 2008 for the remifentanil).

Multi-unit global activity changes in the propofol group has been previously reported^17^. In the current study we used this data as a reference for the remifentanil effect on multi-unit activity and for new frequency domain analysis for both data sets.

### Patients

Candidates considered for this study were patients with advanced PD that met accepted criteria for DBS surgery (idiopathic PD, significant side effects/decrease quality of life and at least 40% decrease in the UPDRS with dopaminergic therapy) and who had been scheduled for bilateral STN DBS. Patients had been preoperatively evaluated by a multidisciplinary group comprising a movement disorder neurologist, neuropsychologist and neurosurgeon. All patients who were found suitable for the DBS procedure were candidates for our experiment unless excluded by one of the exclusion criteria. Exclusion criteria for the study included obstructive sleep apnea, suspected difficult airway and patients where there was concern about the level of cooperation, as well as allergy to eggs/soy or to propofol/remifentanil for the patients that received propofol and remifentanil respectively. Table 1 depicts the study population characteristics, 16 patients in the propofol group (57.6 ± 10.7 years, 12 men) and 12 patients in the remifentanil group (64.2 ± 5.3, 9 men).

**Table 1:** Characteristics of the study populations.

	Gender	Age (years)	Disease duration (years)	LEDD (mg)	ACE	FAB	HDRS
P01	F	60	16	610	91	17	18
P02	M	51	8	1,600	N/A	N/A	N/A
P03	M	62	19	850	89	16	11
P04	M	55	8	1,140	N/A	N/A	N/A
P05	M	60	9	1575	81	17	11
P06	M	52	20	550	88	17	12
P07	M	41	9	1,150	91	16	21
P08	M	54	7	1,150	87	15	8
P09	M	72	10	675	86	13	11
P10	F	61	9	865	75	18	19
P11	M	67	10	675	83	13	11
P12	F	30	10	200	97	N/A	16
P13	M	72	7	1675	92	17	12
P14	M	60	6	200	82	12	25
P15	M	62	12	879	87	18	14
P16	F	63	11	1,230	N/A	N/A	N/A
Average	12 M/4F	57.6	10.7	939	86.8	15.8	14.5
SD		10.74	4.16	455.00	5.65	2.05	4.93
R01	M	61	11	600	80	15	6
R02	M	70	10	1,325	87	16	7
R03	M	73	20	990	N/A	N/A	N/A
R04	F	62	8	940	88	18	8
R05	M	62	17	1,375	81	17	16
R06	F	70	13	450	80	18	12
R07	M	63	13	1,105	88	18	23
R08	M	64	30	3,900	92	17	7
R09	M	61	15	1,299	87	18	5
R10	M	53	3	850	87	17	9
R11	F	66	4	770	91	18	7
R12	M	66	10	1,098	83	18	9
Average	9 M/3F	64.2	12.8	1,225.2	85.8	17.3	9.9
SD		5.3	7.3	889.3	4.2	1.0	5.3
*P *value		0.043	0.374	0.324	0.616	0.036	0.040

Orange and blue colors represent propofol and remifentanil patient groups, respectively. *LEDD*  Levodopa Equivalent Daily Dose (mg) was calculated according to the protocol suggested by Tomlinson et al.^36^. *ACE* Addenbrooke cognitive examination (out of 100), *FAB* Frontal Assessment Battery (out of 18), *HDRS * Hamilton Depression Rating Scale (out of 52). A two tailed T-test comparison between each parameter of both groups showed no significant difference of a *P* value  < 0.01.

### Procedure

Surgical and data collection procedure was as previously reported^17^. Briefly, STN target was identified based on 3 T- magnetic resonance imaging (MRI; T2 weighted axial sequences) imaging using Framelink 5 software (Medtronic). Surgery was performed using the CRW stereotactic frame (Radionics, Burlington, MA, USA). Patients were off dopaminergic medications (> 12 h after last medication).

Using one or two microelectrodes, MER mapping was performed starting 10 mm above the calculated target. When using two microelectrodes, the second advanced in parallel (2 mm anterior/ventral or posterior/dorsal) to the central electrode track (aimed towards the calculated target).

In cases that both hemispheres were operated on, sedatives were used only during the experimental phase that took place during the MER of the second hemisphere due to the possibility that if the sedatives were given during the first hemisphere procedure their effect might continue to the second part of the operation and alter the clinical result. During this phase, a constant electrode position was maintained after either one or both electrodes were inside the STN, preferably in a position where the signal-to-noise ratio recorded was favorable. Patients received an infusion of a sedative substance: either propofol (50 µg·kg^−1^·min^−1^) or remifentanil (0.1 µg·kg^−1^·min^−1^), until patient's sedation level was found to be adequate by the anesthesiologist’s clinical examination (patient was drowsy, but arousable by calling his name or a mild sensory stimulus). Once sedative level was achieved, either propofol or remifentanil administration was stopped, allowing the patient to regain consciousness (awake and follows orders), ending the experimental phase. Subsequently, electrode advancement to complete the MER and localize the ventral border of the STN was resumed. The experimental phase and clinical evaluation were conducted by a board-certified anesthesiologist (either A.R. or D.E.).

### Data acquisition and analysis

Our data acquisition methods have previously been reported elsewhere^17^. We will describe them here briefly: recording of the electrical activity at the tip of the electrode was performed throughout the physiolgical navigation phase (including the experimental phase). Data was obtained at every position in which the electrodes stopped. Recording started 2 s following the stabilization of the electrodes, until the next movement (10–60 s during mapping and 15–25 min during the experimental phase).

Data acquisition was performed using the MicroGuide system (AlphaOmega Engineering, Nazareth, Israel). Neuronal activity was recorded with polyamide-coated tungsten microelectrodes (0.3–0.8 MΩ measured at 1000 Hz, AlphaOmega Engineering, Nazareth, Israel). The recorded signal was amplified either 10,000 or 25,000-fold and band-passed between 250 and 6,000 Hz using a hardware four-pole Butterworth filter. The signal was sampled at 48 kHz (in one case 12 kHz sampling rate was used), by use of a 12-bit A⁄D converter, using a ± 5 V input range.

As previously reported^15^, we used the normalized root mean square (RMS) to estimate the intensity of the multi-unit activity near the tip of the microelectrode. RMS equals the square root of the sum of the squares of differences of each data point from the mean, divided by the number of samples minus one. Normalized RMS was calculated by normalizing the RMS to the RMS recorded during the first 30 s of stable MER prior to STN entry. This normalization minimizes the effect of the electrode impedance (range 0.4–0.8 MΩ at 1000 Hz).

In addition, we performed spectral analysis of the neuronal activity to identify changes in the oscillatory activity. Neuronal data was devided to consecutive 2 s sampling windows with 50% overlap and smoothed using a Hamming window. Power spectrum was calculated using a discrete Fourier transform of the sampling windows to allow evaluation of change in oscillatory activity along time. Significant oscillations were defined as a peak in the average power spectrum, during 3 min of baseline or 3 min of stable sedation, which is larger than 3 standard deviations above the mean of the power between 1 and 100 Hz. For simultaneously recorded pairs of electrodes within the STN we calculated the coherence of the neuronal activity using a 15 s sliding window with 50% overlap and smoothed by a Hamming window. We have decided to use slightly larger windows than for the spectrogram, to compensate for the noisier features of the Coherence function. We used the same definition to define a significant peak in the coherence. All analysis was carried out using custom Matlab R2018a (Mathworks, Natick, MA, USA) routines.

### Statistical analysis

We’ve calculated a sample size of 11 patients for the remifentanil group based on our previously reported propofol results (to indicate an RMS drop from 2.2 to 1.7 with standard deviations of 0.4, using 80% power and alpha of 5%). Thus, we decided to recruit 12 patients (10% over the calculated sample size). We’ve used a paired t-test to compare RMS results between the conditions (baseline and sedated). To compare the effects of the anesthetic drugs on the number of oscillatory electrodes (locations) we transformed the numbers to percentage and compared the percent of oscillatory electrodes in each group before and during sedation using Fisher Exact Test. When not specified otherwise, the statistics presented in this article use mean ± standard error of the mean notation. We’ve considered *P* value smaller than 0.05 to be a significant difference.

## Results

Twelve patients were enrolled to the remifentanil study. We recorded the neuronal activity around the time of remifentanil administration during the MER of the second hemisphere (see Methods) in all these patients allowing a total of 20 recording locations inside the STN (in 8 patients we had 2 electrodes inside the STN and in 4 patients we had only one electrode). These results are compared to previously reported data obtained from 16 patients and 24 locations (8 patients with 2 electrodes and 8 with only one) recorded around the time of propofol administration^16^, and to new frequency domain analysis of this data.

### Level of consciousness

Sedation level was assessed by clinical evaluation. Our purpose was light sedation, defined as a drowsy patient, arousable by calling his name or a light tap on the shoulder. We were able to achieve this goal using with both propofol and remifentanil. Stable sedation was achieved within a few minutes with both drugs. However, in 3 of the remifentanil patients we had to increase the dose to 0.15–0.18 µg kg^−1^·min^−1^ in order to achieve this level of sedation prolonging the time it took to achieve stable sedation. In one case we used a small remifentanil bolus which dramatically shortened the time to reach good sedation.

### Neuronal spiking activity

**Figure 1:**
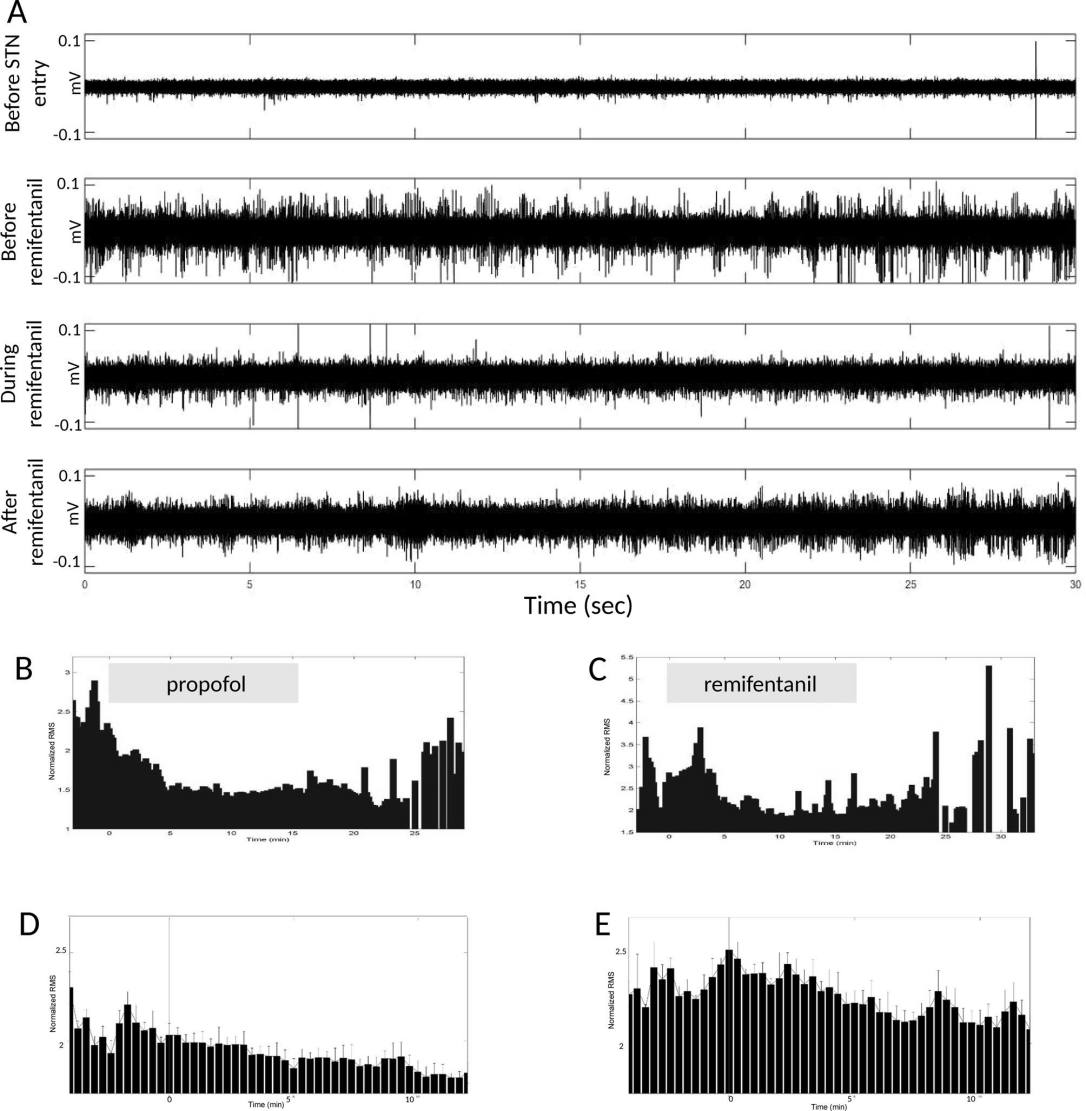
Effects of sedation on the global spiking activity in the STN. **A**: Raw data examples from a single trajectory. Top: before STN entry (presumably in white matter). Second line: inside the STN, before remifentanil administration. Third line: inside the STN, at the same location during remifentanil administration (patient sedated to the desired level). Bottom: inside the STN, at the same location, after remifentanil administration stopped, patient awake and responsive. All traces are 30 s long and presented with the same y scale. **B**–**C**: Examples of normalized root mean square (RMS) changes during propofol (**B**) and remifentanil (**C**) administration. X-axis = time (min); Y-axis = normalized RMS. Gray bars above the graphs indicates duration of drug administration. **D**–**E**: Population normalized. RMS. Aligned to the beginning of propofol (**D**) or remifentanil (**E**) administration. X-axis = time (min); Y-axis = normalized RMS. Subplots B and D are modified from Raz et al. Anesth Analg 2010; 111:1,285–9 for easy comparison of propofol and remifentanil effects.

Figure 1A shows an example of raw data recorded along an electrode trajectory: first, before STN entry, and then inside the STN before remifentanil administration, during administration and after administration was stopped (at the same location). Our analysis demonstrates that spiking activity (measured by the normalized RMS of the 250–6000 Hz band-passed data, and include both high amplitude and background spiking) dropped following administration of either propofol or remifentanil (see Fig. 1B–E). Unlike propofol which significantly depressed STN activity as manifested by a 23% drop of the RMS^17^, remifentanil effect was moderate and decreased the normalized RMS from 2.3 ± 0.1 to 2.1 ± 0.1 (8.7%, p < 0.001, paired t-test, see Fig. 1D, E).

### Oscillatory neuronal activity and coherence

**Figure 2:**
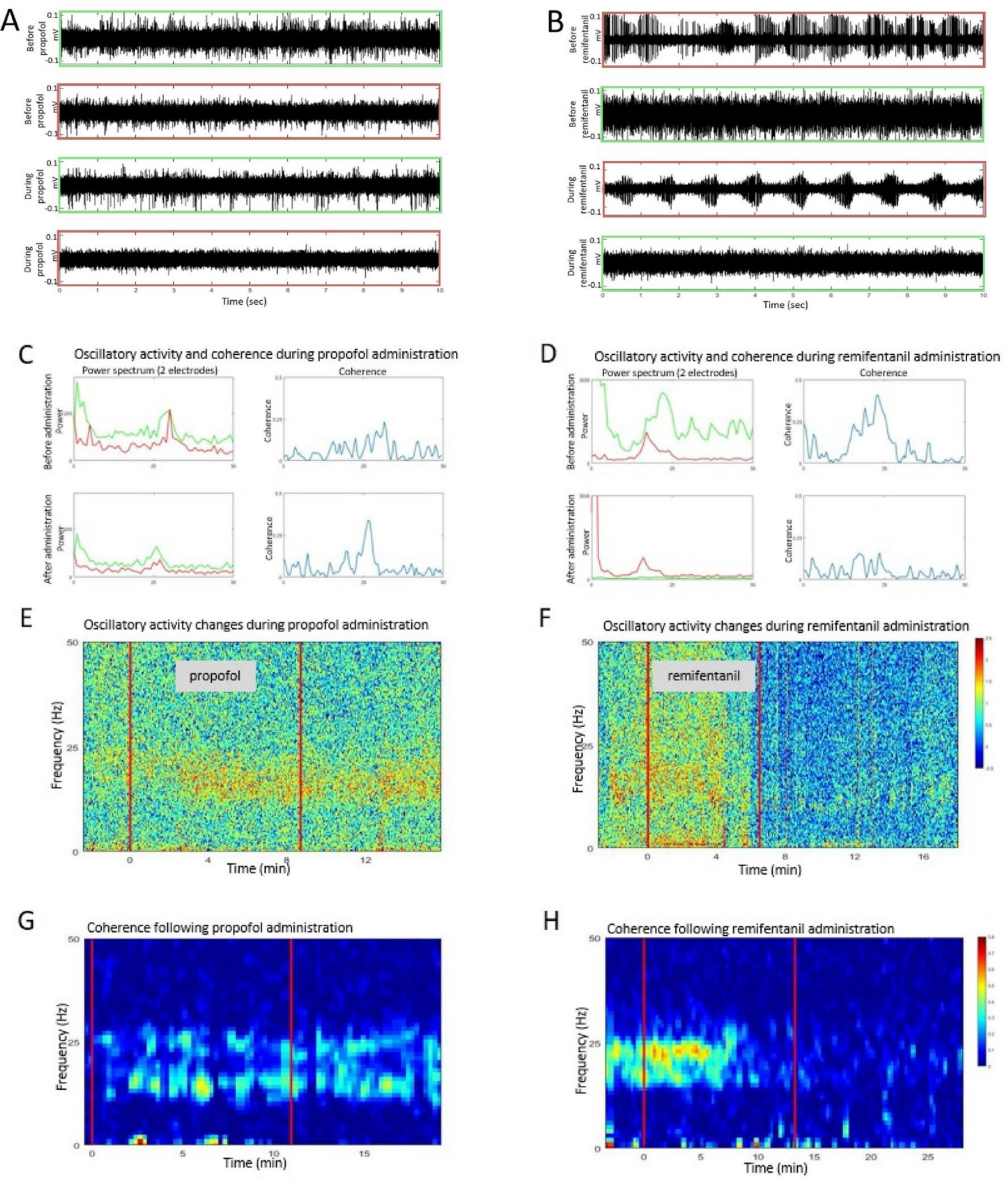
Effects of sedation on oscillatory activity in the STN. Green and red frames (**A**, **B**) represent two cells which are also depicted in figures C and D for propofol and remifentanil, respectively. **A:** Raw data examples from two distinct cells. Top row: First cell, before propofol administration. Second row: Second cell, before propofol administration. Third row: First cell, during propofol administration (patient sedated to the desired level). Bottom row: Second cell, during propofol administration (patient sedated to the desired level). All traces are 10 s long and presented with the same y scale. **B**: The same as A, for remifentanil administration. **C**: Average power spectrum (left) and coherence (right) of 30 s of neural (multi-unit) activity in a single location measured from one patient before (top) and during (bottom) administration of propofol. It can be seen that no reduction of the beta power oscillations or coherence was observed. **D**: Same as C but with administration of remifentanil, measured from a different patient. It can be seen that one of the cells lost its’ oscillatory behavior, and the previously robust synchronization disappeared. **E**: Power spectrum of the multi-unit activity recorded by a single electrode measured around the time of propofol administration. It can be seen oscillatory activity in the beta power increased during propofol sedation. **F**: Same as **E**, but around the time of remifentanil administration. It can be seen that significant reduction in the beta power was observed during remifentanil administration. **G**: Coherence of the mutli-unit activity of two simultaneously recorded electrodes measured around the time of propofol administration. It can be seen that there is a significant coherence between the two electrodes which is maintained under sedation with propofol. **H**: Same as G but during administration of remifentanil. It can be seen that there is a significant coherence between the two electrodes which is depressed after remifentanil administration. For **E**–**H**, x-axis = time (min); y-axis = frequency (Hz) and color code represents power (**E**, **F**) or coherence (**G**, **H**). Time of sedative drug administration (propofol—left column, remifentanil—right column) is marked by a vertical line.

Previous studies demonstrated typical oscillatory activity in the beta frequency range (13–30 Hz) that dominate the dorsolateral STN of PD patients^4,15,16^. As expected, we encountered this oscillatory activity in the STN before administrating any sedation. Previous reports have also demonstrated that these oscillations tend to be synchronized if the two electrodes are located within the dorsolataeral oscillatory region^21^. We had only a few cases where we had two electrodes simultaneously located within the dorsolateral oscillatory region during the experimental phase, making it difficult to draw conclusions. However, in those cases that we did have such pairs, we often observed coherence in the beta range between the electrodes before the administration of the sedative agents (see Fig. 2).

Of 24 STN electrodes (locations) recorded in the propofol group, we observed robust oscillatory activity in the beta range on 8 electrodes. Following propofol administration, this increased to 10 electrodes (see example in Fig. 2A). In two out of eight pairs recorded, we observed significant coherence between the two electrodes. This increased to 3 pairs after administration of propofol (see examples in Fig. 2C, E).

Of 20 STN electrodes recorded in the remifentanil group, we observed oscillatory activity in the beta range in 5 electrodes. Following remifentanil administration, oscillatory activity stopped in 4 electrodes remaining significant in only one (see example in Fig. 2B). We had only one pair with significant coherence before the sedation. Following remifentanil administration the coherence of this pair significantly dropped in parallel to the oscillatory activity recorded by one of the electrodes and to the clinical effect (see examples in Fig. 2D, F).

We used the percentage of oscillatory electrodes to compare the amount of oscillatory activity between the two anesthetics (see Methods), there was a significant difference between the effect of the two drugs (Fisher Exact = 0.0002, *P* < 0.01, Fisher Exact test). The difference of the pair-wise coherence was not statistically significant, but the number is too small to draw conclusions. It should also be noted that the depression of the oscillatory activity and synchronization following remifentanil was prolonged, and the oscillatory activity and coherence did not recover for a while even after the patient was fully awake clinically.

## Discussion

We recorded the neuronal activity at a constant STN location under propofol and remifentanil sedation. Remifentanil decreased the global spiking activity to a much lesser extent than propofol. Beta oscillations and synchronization were not significantly affected by propofol, while remifentanil seems to diminish the oscillations and the synchronization.

Patients with advanced PD may be offered DBS surgery to improve their symptoms^1,2^. This surgery can be done under either general or local anesthesia^7,22^. A recently published meta-analysis of DBS for PD compared the outcomes of these two options^23^. The results demonstrated equivalence in terms of motor outcome. Local anesthesia with an awake patient was superior to general anesthesia regarding treatment related side effects as measured by the unified Parkinson’s disease rating scale (UPDRS). However, general anesthesia was associated with lower rates of complication, such as intracerebral hemorrhage and infections. Another systematic review showed no significant differences in the postoperative UPDRS regarding clinical outcomes^24^. Thus, the preferred anesthetic approach, local versus general, is not yet determined.

We have previously shown that STN neuronal activity returned to baseline shortly after propofol administration ceased^17^. Thus, propofol can safely be used until shortly (10–15 min) before electrophysiological mapping begins, reducing patient anxiety and discomfort in the initial phase of surgery. In the current study, we observed the effects of remifentanil administration on the fidelity of MER and compared it to the effects of propofol. Remifentanil significantly reduced the level of neuronal activity (RMS) but to a much lesser extent than propofol, this relatively small drop (9%, compared to 23% with propofol) would probably allow a reasonable identification of the STN borders using RMS guidance. On the other hand, remifentanil diminished the oscillatory activity and synchrony observed in the STN, while propofol was not associated with any significant reduction of oscillations or synchrony.

As RMS of 250–6000 Hz band-passed signal is probably directly related to the cumulative firing rates of neurons near the tip of the electrode, our results seem to be in conflict with previously published results by McIver et al.^25^ which demonstrated no effect of propofol or remifentanil on the firing rates and pattern of STN neurons. It should be noted however that McIver et al. used a bolus administration, while we used continuous infusion titrated to the clinical response. Thus, our doses tended to be higher (e.g. for the propofol, on average we’ve used double the dose reported by McIver et al.). The different results suggest that the effect may be dose and/or arousal level dependent. This is supported by Moll et al.^20^ who demonstrated that titration to a lower dose decreased the changes seen in the STN activity pattern, while increasing the dose augmented the differences between local and general anesthesia, with either propofol or remifentanil.

Remifentanil is a synthetic opioid analogue affecting mostly Mu-opiate receptors^26^.

In a normal healthy brain Mu-opiate receptors are activated by enkephalin, an endogenous opioid that is secreted by the indirect-pathway striatal neurons as a co-transmitter along with gamma-aminobutyric acid (GABA)^27,28^. The degeneration of dopaminergic neurons in PD patients results in decreased inhibition of the indirect pathway, leading to excess GABA and enkephalin release in the globus pallidus externa^29,30^. Studies using 1-methyl-4-phenyl-1,2,3,6-tetrahydropyridine (MPTP) to induce murine^30^ and primate^31,32^ models of parkinsonism demonstrated increased levels of enkephalin expression in the striatum, parallel with the degeneration of nigrostriatal neurons. Our results showed that remifentanil significantly decreased oscillatory activity and synchrony within the STN, while propofol (a GABA_A_ agonist) administration that caused a similar degree of sedation, had a stronger effect on the global spiking activity^17^ but minimal effect on the oscillation and synchronization. These findings might show a possible connection between opiate receptors and the changes in synchronization and oscillation seen in PD. Further research should be conducted to reveal whether these effects are dose dependent and if there is a direct connection between activation of opiate receptors and basal ganglia synchronous oscillations.

### Limitations

A major limitation that should be acknowledged is the lack of an objective tool to assess the depth of anesthesia, such as EEG monitoring during the MER procedure. Therefore, the changes seen in the STN data might have been due to the different arousal state that could not be detected in a subjective manner. Another limitation of this study is that spike sorting was not performed, thus normalized RMS data may not be informative enough to draw conclusions at the level of a single cell. Finally, we have not tested the effect of the sedation drugs on the clinical symptoms of the patients. Given the reports of remifentanil induced muscle rigidity^33–35^, this aspect should be further explored during future studies. Note that Table 1 includes data that was retrospectively traced from patient files. Thus, some of the patient’s clinical data is missing.

## Conclusions

Remifentanil may be better than propofol with regards to RMS identification of the STN borders, but the changes observed in oscillatory activity may interfere with identification of the dorsolateral oscillatory region. This may be significant, as this region seems to be the optimal target for electrode implantation^4^. Thus, it may be prudent to reduce or stop remifentanil administration long enough prior to the anticipated starting of MER.
